# Lack of Associations between Female Hormone Levels and Visuospatial Working Memory, Divided Attention and Cognitive Bias across Two Consecutive Menstrual Cycles

**DOI:** 10.3389/fnbeh.2017.00120

**Published:** 2017-07-04

**Authors:** Brigitte Leeners, Tillmann H. C. Kruger, Kirsten Geraedts, Enrico Tronci, Toni Mancini, Fabian Ille, Marcel Egli, Susanna Röblitz, Lanja Saleh, Katharina Spanaus, Cordula Schippert, Yuangyuang Zhang, Michael P. Hengartner

**Affiliations:** ^1^Department of Reproductive Endocrinology, University Hospital ZürichZurich, Switzerland; ^2^Department of Psychiatry, Social Psychiatry and Psychotherapy, Medical School HannoverHannover, Germany; ^3^Department of Computer Science, Sapienza Università di RomaRome, Italy; ^4^Center of Competence in Aerospace, Biomedical Science and Technology, Lucerne University of Applied Sciences and ArtsLucerne, Switzerland; ^5^Computational Systems Biology Group, Zuse InstituteBerlin, Germany; ^6^Institute of Clinical Chemistry, University Hospital ZürichZurich, Switzerland; ^7^Department of Gynaecology and Obstetrics, Hannover Medical SchoolHanover, Germany; ^8^Department of Applied Psychology, Zurich University for Applied Sciences (ZHAW)Zurich, Switzerland

**Keywords:** hormones, cognition, menstrual cycle, working memory, attention, cognitive bias, estrogen, progesterone

## Abstract

**Background:** Interpretation of observational studies on associations between prefrontal cognitive functioning and hormone levels across the female menstrual cycle is complicated due to small sample sizes and poor replicability.

**Methods:** This observational multisite study comprised data of *n* = 88 menstruating women from Hannover, Germany, and Zurich, Switzerland, assessed during a first cycle and *n* = 68 re-assessed during a second cycle to rule out practice effects and false-positive chance findings. We assessed visuospatial working memory, attention, cognitive bias and hormone levels at four consecutive time-points across both cycles. In addition to inter-individual differences we examined intra-individual change over time (i.e., within-subject effects).

**Results:** Estrogen, progesterone and testosterone did not relate to inter-individual differences in cognitive functioning. There was a significant negative association between intra-individual change in progesterone and change in working memory from pre-ovulatory to mid-luteal phase during the first cycle, but that association did not replicate in the second cycle. Intra-individual change in testosterone related negatively to change in cognitive bias from menstrual to pre-ovulatory as well as from pre-ovulatory to mid-luteal phase in the first cycle, but these associations did not replicate in the second cycle.

**Conclusions:** There is no consistent association between women's hormone levels, in particular estrogen and progesterone, and attention, working memory and cognitive bias. That is, anecdotal findings observed during the first cycle did not replicate in the second cycle, suggesting that these are false-positives attributable to random variation and systematic biases such as practice effects. Due to methodological limitations, positive findings in the published literature must be interpreted with reservation.

## Introduction

In the scientific literature, female sex hormones and the menstrual cycle have been linked to cognitive performance (Farage et al., 2008; Sherwin, 2012). The main tenet of this work is that sexually dimorphic cognitive skills that favor men (i.e., visuospatial tasks) are improved during menstrual phases with low estrogen and/or progesterone, while skills that favor women (i.e., verbal tasks) are improved during phases of high estrogen/progesterone. This work has been extended to test prefrontal cortex functions and along these lines it has been suggested that estrogens may be significantly involved in attention and working memory (e.g. Solis-Ortiz and Corsi-Cabrera, 2008; Hatta and Nagaya, 2009; Jacobs and D'Esposito, 2011). However, a variety of studies found no or inconsistent associations, as recently detailed in a comprehensive review and synthesis of the literature (Sundstrom Poromaa and Gingnell, 2014). Moreover, there are growing concerns that various positive associations reported in the literature could be true null associations, that is, methodological artifacts and chance findings (Ioannidis, 2005; Rosmalen and Oldehinkel, 2011; Ferguson and Heene, 2012; Hengartner, 2017). In support of this notion it has been demonstrated that due to scientific biases, first, false-positive findings are ubiquitous, second, that inflated effect sizes are common and, third, that the reproducibility of results is generally low in psychological and biomedical research (e.g., Kriegeskorte et al., 2009; Prinz et al., 2011; Ritchie et al., 2012; Button et al., 2013; Macleod et al., 2014; Open Science Collaboration, 2015; Muller et al., 2017). The ongoing question is therefore, whether the fluctuations of female sex hormones across the menstrual cycle really influence attention and working memory in a consistent way or whether these positive findings are spurious false-positives due to scientific fallacies and methodological biases.

As recently reviewed, most published studies failed to find meaningful and consistent associations between hormones and cognitive functioning in women (Sundstrom Poromaa and Gingnell, 2014). One reason for the inconsistencies between findings is that cross-sectional studies (e.g., Halari et al., 2005; Hampson and Morley, 2013) do not allow for drawing stringent conclusions and that many longitudinal studies relied on only two measurements (e.g., Maki et al., 2002; Schoning et al., 2007; Hatta and Nagaya, 2009; Jacobs and D'Esposito, 2011). An additional major source of bias within the field are the utterly small sample sizes, which commonly include less than 30 women (e.g., Maki et al., 2002; Schoning et al., 2007; Jacobs and D'Esposito, 2011), and in some highly-cited studies even less than 10 (e.g., Hausmann et al., 2000; Solis-Ortiz et al., 2004; Solis-Ortiz and Corsi-Cabrera, 2008). As comprehensively reviewed by Button et al. (2013), underpowered small samples substantially undermine the reliability of research findings by producing severely inflated effect sizes and both false-positive and false-negative results. Various studies also made speculative inferences from cycle phase on hormone levels without actually reporting a correlation between hormones and cognition (e.g., Rosenberg and Park, 2002; Solis-Ortiz et al., 2004; Solis-Ortiz and Corsi-Cabrera, 2008). Such inferences are problematic because inter-individual variance in hormone levels at particular phases of the cycle is tremendous (Sundstrom Poromaa and Gingnell, 2014). That is, it must not be concluded that inter-individual differences between cognitive functioning and cycle phase is causally related to estrogen simply because, for instance, estrogen is *on average* higher around ovulation than premenstrually, since a substantial portion of women have higher estrogen levels premenstrually than at ovulation. A stringent test would be to examine whether intra-individual change from ovulatory to premenstrual phase relates to changes in cognitive functioning. If not, then a causal relationship is unlikely, but unfortunately intra-individual change has hardly been considered in this field. Of major concern are also reporting and publication biases, which prevent the dissemination of negative findings and which lead to severe overestimation of associations in the published literature (Ioannidis et al., 2014). Thus, there is apparently a gap between the actual meaning of research findings and conclusions drawn in some narrative reviews and original studies. In view of the methodological shortcomings in the literature on associations between hormones and cognitive abilities detailed above, methodologically sound observational studies are necessary.

To come at reliable and valid estimates, larger samples (i.e., preferably *n* > 50) more fine-grained designs (i.e., 4 repeated measurements across the cycle), more sophisticated statistical modeling (examination of intra-individual change) and replication of results (i.e. using data from a second cycle) are required. We thus postulate the following four propositions for a reliable and meaningful finding: Firstly, there needs to be evidence for significant differences in cognitive functioning across the cycle (inter-individually and specifically intra-individually). Secondly, between-subject differences in hormone levels must relate to differences in cognitive abilities at specific cycle phases. Thirdly, these effects need to replicate in significant associations between intra-individual change in hormone levels and cognitive abilities across the cycle. Fourthly, significant effects must replicate in data obtained from a second menstrual cycle to exclude practice effects and false-positive chance findings. The aim of the present work was to critically examine, whether prefrontal cortex functions such as working memory, attention and cognitive control relate to serum hormone levels. A recent review suggested that these cognitive functions may correlate positively with estrogen and progesterone (Sundstrom Poromaa and Gingnell, 2014), but due to the inconsistencies and methodological limitations in the literature on associations between cognitive functioning and hormones detailed above, we did not a priori postulate specific hypotheses.

## Methods

### Participants and design

The study was designed as a prospective observational study investigating serial measurements of hormonal and neurocognitive parameters in healthy women and women with endocrine disorders aged 18–40 years in up to two menstrual cycles. Data were collected from 88 menstruating women. Of those women, 58 presented no endocrinological pathology, 13 were diagnosed with endometriosis, 16 with polycystic ovary syndrome (PCOS) and one woman with hyperprolactinemia. Also, 12 women presented with obesity (defined as BMI > 30.0). Altogether 50 women were recruited at the Department of Psychiatry, Social psychiatry and psychotherapy, Medical School Hannover, Germany, and 38 women at the Clinic for Reproductive Endocrinology, University Hospital Zurich, Switzerland. All women with endometriosis, PCOS or hyperprolactinemia were recruited in Zurich. Word of mouth, direct invitation of eligible women in the consultations of the Clinic for Reproductive Endocrinology, University hospital Zurich, Switzerland and by gynecologists specialized in gynecological endocrinology as well as advertisement on the hospital and university boards were used for recruitment. A total of 68 women were re-assessed during a second menstrual cycle (Hannover: *n* = 47; Zurich: *n* = 21). For every completed cycle, participants received 600 Swiss Francs (at Zurich study site) or 500 Euros (at Hannover study site). As assessed before the first index cycle, the mean age was 30.2 years (*SD* = 5.5) and ranged from 20 to 40 years. The mean BMI was 25.0 (*SD* = 5.4) and ranged from 17.7 to 45.7. A total of *n* = 31 (34.4%) were married and *n* = 27 (30.0%) had children. Finally, *n* = 27 (30.3%) had a university degree. This study followed the guidelines of the World Medical Association Declaration of Helsinki 1964, updated in October 2013 and was conducted after approval by the Ethics Committee of Hannover and Zurich for investigations involving human subjects. All participants provided written informed consent. Women were compensated for their expenditures associated with study participation. The study has been registered in clin.trial.gov (NCT02098668).

During a baseline visit women were interviewed to verify inclusion and exclusion criteria and a physical examination was performed to exclude medical conditions which might influence hormone levels or cognitive performance except for endometriosis, PCOS or hyperprolactinemia. Women were excluded if they were using oral contraceptives, had been pregnant or breastfeeding within the past 6 months, were using medication or had surgery which might interfere with endocrine parameters, had severe psychiatric or general diseases, worked irregular shifts, had menstrual or ovulation disorders except those investigated in the study (endometriosis, PCOS and hyperprolactinemia) and if they showed any additional abnormality in hormonal parameters (LH, FSH, estradiol, progesterone, testosterone, prolactin, fasting glucose, fasting insulin, thyroid stimulating hormone and in Zurich also anti-Millerian hormone) taken cycle day 2–5 in the cycle following the clinical screening examinations.

### Hormone measurements and assays

For each woman with a cycle length of 28 ± 4 days a series of 8 measurements of hormonal parameters was scheduled at predefined days of the cycle (at cycle day 4, 7, 9, or 10, 12, 13, 17, 21, 28). At the first measurement and within 3–5 days prior to the earliest anticipated day of ovulation based on the menstrual history of the six previous cycles a first transvaginal ultrasound was performed to exclude any cysts interfering with the menstrual cycle. A second ultrasound was performed around cycle day 11 to measure follicular development in order to place the pre-ovulatory measurement as precise as possible. When no dominant follicle could be demonstrated in the second ultrasound control additional measurements were performed in 4–5 day intervals until follicular development could be confirmed or cycle day 30 was reached. Ovulation tests based on urine LH measurements (Evial Ovulationstest Midstream, Inopharm GmbH, Muri, Switzerland and Clearblue digital Ovulationstest, SPD Swiss Precision Diagnostics GmbH, Geneva, Switzerland) were used to confirm the day of ovulation. These tests were started either 5 days prior to the earliest ovulation based on the previous 6 cycles or when a 14 mm follicle was seen through transvaginal ultrasound. At each visit blood samples were collected for hormonal assessment between 7.00 and 10.00 a.m. At four time points e.g., cycle day 2–5 (menstrual), pre-ovulatory, mid-luteal and premenstrual the participants took neuropsychological tests in addition to blood sampling.

In Hannover blood samples were initially frozen at −30°C and then stored at −80°C. In Zürich blood samples were sent to the laboratory immediately after the sample was collected in the morning. To avoid bias due to different laboratory procedures all samples were analyzed by the laboratory in Zürich. Estradiol was measured using electrochemiluminescence immunoassays ECLIA (Elecsys® Estradiol II) based on polyclonal antibody (Roche Diagnostics GmbH, Penzberg, Germany) with a functional assay sensitivity of 44 pmol/L and a coefficient of variation (CV%) of less than 7.7%. From January 15th 2015, the ECLIA (Elecsys® Estradiol III) based on monoclonal antibody (Roche Diagnostics GmbH, Penzberg, Germany) with a functional assay sensitivity to 91.8 pmol/L (25 pg/mL) and CV% to less than 3.36% was applied. The measurement of LH, FSH, progesterone, testosterone, TSH, and prolactin were performed using electrochemiluminescence immunoassays (ECLIA) applied on Cobas e-602 immunoassay autoanalyzer (Roche Diagnostics GmbH, Penzberg, Germany).

The functional analytical assay sensitivity for LH, FSH, progesterone, testosterone, TSH, and prolactin was 0.1 IU/L, 0.1 mIU/L, 0.48 nmol/L, 0.416 nmol/L, 0.014 mIU/L, and 1.00 μIU/mL (0.047 ng/mL), respectively. Total imprecision (intra-assay and inter-assay) of each assay was assessed by measuring 20 replicates of quality control samples over 20 days. Total imprecision expressed as coefficient of variation (CV%) for LH, FSH, progesterone, testosterone, TSH, and prolactin was less than 2.2, 2.1, 5.1, 3.9, 2.5, and 1.3 respectively. All analyses described in this section were performed at the Institute of Clinical Chemistry, University Hospital Zurich. For all methods, external quality controls were carried out at regular intervals by the society for promoting quality assurance in medical laboratories (INSTAND, Duesseldorf, Germany) and Reference Institute for Bioanalytics (RfB, Bone, Germany).

### Neuropsychological tests

Cognitive tests were performed using a standardized, validated, computer-assisted test system developed by Candit.com (CANDIT: Computer Assisted Neuropsychological Diagnostics and Therapy). The neuropsychological tests were performed on a touch screen computer; the same model was used in Hannover and Zurich. Ten CANDIT tests were performed by each subject in total and the overall test time was approximately 40 min. The test categories included attention (comprising Cancellation Screen Short, CPT Visual Short, and Divided Attention Bimodal Task), visual memory (Blockspan forwards and backwards) and executive functions tests (Cognitive Bias Test). These tests (and their adaptations) were applied in previous research on associations between cognitive functioning, hormones and cycle phases (e.g., Mordecai et al., 2008; Solis-Ortiz and Corsi-Cabrera, 2008; Hampson and Morley, 2013). The other 5 tests were created or adapted from standardized CANDIT tests and these categories ranged from food cravings (Food Craving), dyadic coping (Implicit Dyadic Coping), reactions to sexual stimuli (Emotional Cognitive Bias Test and Rating of Sexual Stimuli) and emotions (Situation Rating). Due to parsimony and in order to avoid redundancy and false-positives due to multiple testing, for the present paper we randomly chose one test out of the three attention tests, that is, the divided attention test. Food craving, implicit dyadic coping, as well as sexual stimuli and emotion tests were excluded from the present study because they do not assess prefrontal cognitive abilities. Participants were placed in a quiet room to complete all the tests with a trained study staff member present to explain the tests and answer any questions that might arise during the test.

The Blockspan test is a well-established tool to investigate visuospatial working memory (Vandierendonck et al., 2004; Doucet et al., 2013) by requiring forward and backward recall of path presentations. This test is also known as the Corsi blocks task. In short, for the block task a set of 9 identical blocks is presented on a monitor. Upon presentation of a series of blocks, which change their color in a consecutive order, a representation of the path has to be constructed and maintained in visual-spatial working memory. The sequence then has to be reproduced in the same (phase 1) or in reverse order (phase 2). To reproduce the reverse order executive control is required (Vandierendonck et al., 2004; Alvarez-Moya et al., 2011).

The Cognitive Bias Test (CBT) is a multiple choice procedure designed by Goldberg et al. (1994) as a bias (preference) to evaluate complex cognitive functions. The CBT entails designs characterized along five binary dimensions: shape (circle/square), color (red/blue), number (one/two identical components), size (large/small), and contour (outline/filled with a homogeneous color). Study participants have to rate similarity between two items. The items are on different levels of difficulty and presented twice in different vertical positions to the study participant. Thus, 32 stimuli can be generated, and a “similarity index” computed between any two stimuli, ranging from 5 (identical) to 0 (differing along all five dimensions). The “similarity indices” between targets and subject's choices are summed across trials (Goldberg et al., 1994). In the present study we used correct responses as the outcome, that is, higher scores on the CBT indicate better cognitive control.

The Divided Attention Bimodal Task investigates the ability to control visual and auditory stimuli simultaneously, hence, divided attention. The study participant has to react to predefined visual as well as auditory cues as quick as possible. For each fitting visual cue a specific tab has to be pressed with the left hand and for each fitting auditory cue a specific tab has to be pressed with the right hand. The test includes three test phases, each including a series of 35 items and takes about 5.15 min to be performed (Parasuraman, 1998).

### Statistical analysis

The associations between repeated measures of cognitive functioning and hormone levels were estimated using Generalized Estimating Equations (GEE). These statistical models were introduced to fit regression analyses that account for within-subject correlation, which is an inherent part of longitudinal studies that rely on repeated outcome measures (Zeger et al., 1988). GEE are considered state of the art for longitudinal data analysis and superior to repeated measures ANOVA due to their psychometric properties (Ballinger, 2004; Gibbons et al., 2010). GEE use all available data and impute missing values under the assumption of Missing Completely at Random (MCAR). Repeated measures of cognitive test scores were successively entered as the outcome variables and the hormone measures separately as predictor variables. Because all cognitive test scores were approximately normally distributed, we fitted all models with normal distribution and identity link-function. The within-subject covariance was specified with the “unstructured” correlation type to avoid having any constraints on the covariance structure and a robust sandwich estimator was used to reduce the effects of outliers and influential observations. In addition to inter-individual differences at specific cycle phases, we computed a longitudinal intra-individual change model (Twisk, 2003). In such a model, only within-subject effects are considered by including relative change values between consecutive measurements of both the outcome variable and the predictor variable instead of absolute values for each time-point. Following Twisk (2003), in these change models the covariance structure was specified as “independent.” Results were reported with standardized regression coefficients (β) and their standard errors (SE). In bivariate analyses these correspond to an effect size r. We used two-tailed significance testing and Bonferroni correction for multiple testing was applied to reduce the α-error rate. The Bonferroni-corrected significance level was α = 0.008. All analyses were performed with SPSS 23 for Windows.

## Results

**Figure 1:**
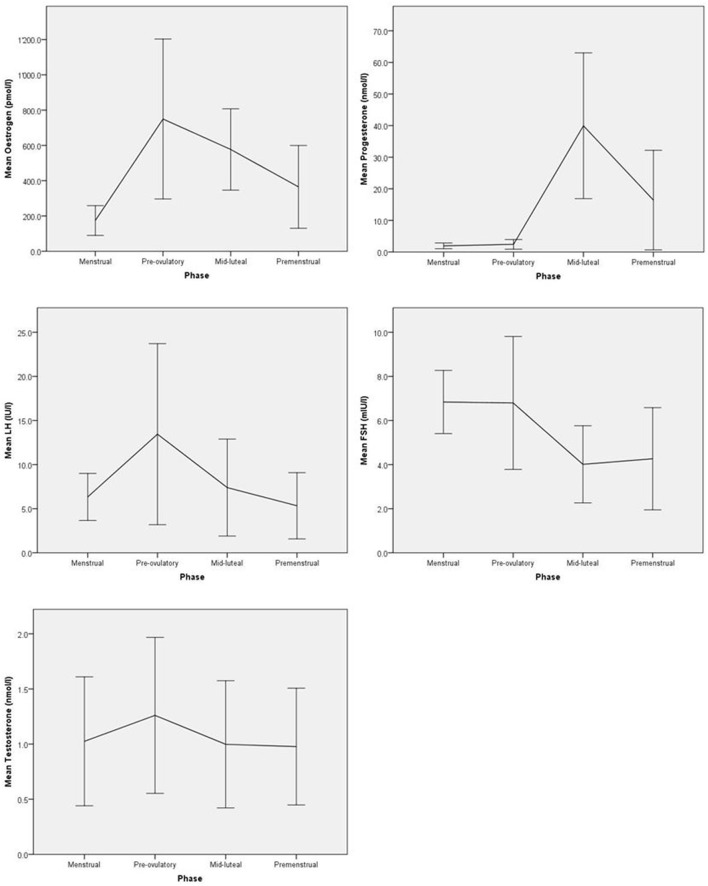
Mean hormone levels with standard deviations across the first menstrual cycle.

Distributions of hormone levels were carefully screened using boxplots and extreme outliers (defined as a value 3 times higher than the 75th percentile) were excluded from the analysis. Such extreme hormone levels concerned 1–2 women per hormone assessed. The fluctuating levels of hormones across the menstrual cycle are indicated in Figure 1. A detailed account of the exact number of assays at each cycle phase, range, means and standard deviations for all hormone measures is provided in the Supplementary Table 1. A total of *n* = 4 and *n* = 3 women, respectively, had an anovulatory cycle at first and second menstrual cycle. Excluding these women did not alter the results reported below; therefore they were included in the analysis.

There was evidence for significant variation in mean test scores across the first cycle for cognitive bias and divided attention and for blockspan forwards across the second cycle (see Table 1). A detailed examination of intra-individual change in cognitive functioning across both cycles is provided in Table 2. Again we found significant effects in cognitive bias and divided attention across the first cycle, indicating that scores in cognitive bias increased intra-individually from early to late follicular phase, whereas scores in divided attention increased from early to late follicular phase as well as from late follicular to early luteal phase. Adjusting for obesity or endocrinological disorders did not alter the results. Moreover, neither obesity, nor endometriosis or PCOS were significantly associated with cognitive functions in the multivariable models. During the second cycle, there was a significant change from early to late follicular phase in cognitive bias and from early to late luteal phase in blockspan forwards.

**Table 1:** Prefrontal cognitive test scores across two menstrual cycles.

**Cognitive Test**	**Measurement occasion**				**Model effect**
	**Menstrual**	**Pre-ovulatory**	**Mid-luteal**	**Premenstrual**	
	**Mean (SE)**	**Mean (SE)**	**Mean (SE)**	**Mean (SE)**	***p***
**CYCLE 1**					
Blockspan forwards	5.82 (0.17)	6.07 (0.13)	6.14 (0.13)	6.15 (0.14)	0.198
Blockspan backwards	5.97 (0.08)	5.91 (0.06)	5.91 (0.08)	5.95 (0.08)	0.801
Cognitive bias	33.62 (0.34)	34.77 (0.32)	34.55 (0.35)	34.36 (0.38)	0.006
Divided attention	95.41 (0.53)	96.95 (0.67)	98.54 (0.59)	97.96 (0.64)	<0.001
**CYCLE 2**					
Blockspan forwards	6.07 (0.14)	6.30 (0.16)	6.13 (0.14)	6.60 (0.12)	0.001
Blockspan backwards	5.93 (0.08)	5.73 (0.12)	5.96 (0.10)	5.66 (0.12)	0.059
Cognitive bias	34.02 (0.56)	34.99 (0.45)	35.02 (0.53)	34.94 (0.52)	0.050
Divided attention	98.12 (0.65)	97.82 (0.73)	98.34 (0.76)	98.00 (0.91)	0.829

**Table 2:** Mean intra-individual changes in prefrontal cognitive test scores across two menstrual cycles.

**Cognitive Test**	**Measurement occasion**			**Model effect**
	**T1 thru T2**	**T2 thru T3**	**T3 thru T4**	
	**Mean (SE)**	**Mean (SE)**	**Mean (SE)**	***p***
**CYCLE 1**				
Blockspan forwards	0.30 (0.18)	0.00 (0.12)	0.02 (0.14)	0.424
Blockspan backwards	−0.04 (0.09)	0.02 (0.09)	0.02 (0.10)	0.861
Cognitive bias	1.27 (0.33)^*^	−0.24 (0.30)	−0.11 (0.29)	0.002
Divided attention	2.11 (0.67)^*^	1.21 (0.60)^*^	−0.59 (0.75)	0.023
**CYCLE 2**				
Blockspan forwards	0.24 (0.20)	−0.11 (0.15)	0.58 (0.14)^*^	0.008
Blockspan backwards	−0.10 (0.12)	0.15 (0.12)	−0.24 (0.13)	0.177
Cognitive bias	0.77 (0.35)^*^	0.13 (0.33)	0.02 (0.32)	0.273
Divided attention	0.58 (0.59)	0.66 (0.56)	−0.27 (0.68)	0.523

* *Change score significantly different from zero*.

*T1, Menstrual phase; T2, pre-ovulatory phase; T3, mid-luteal phase; T4, premenstrual phase*.

*Statistically significant parameter estimates are highlighted in bold face*.

Associations between inter-individual differences in cognitive ability and hormone levels across the first cycle are reported in Table 3. Significant parameters were only considered when the overall model effect was statistically significant at α = 0.05. Testosterone related significantly across measurements to blockspan backwards (*p* = 0.031), but when Bonferroni correction for multiple testing was applied, no single parameter met statistical significance (all *p* > 0.008). FSH related negatively to divided attention at premenstrual phase of the first cycle (β = −0.29, *p* = 0.003). That association held when additionally adjusted for overweight, endometriosis or PCOS. Using data from the second menstrual cycle, we opted to replicate that association. However, at premenstrual phase of the second cycle, FSH was unrelated to divided attention, that is, the association did not replicate (β = −0.01, *SE* = 0.11, *p* = 0.895).

**Table 3:** Associations between inter-individual differences in prefrontal cognitive test scores and hormone levels across the first cycle.

**Cognitive Test**	**Hormones**	**Measurement**			
		**Menstrual**	**Pre-ovulatory**	**Mid-luteal**	**Premenstrual**
		**β (SE)**	**β (SE)**	**β (SE)**	**β (SE)**
Blockspan forwards	Estrogen	0.04 (0.15)	−0.10 (0.11)	−0.10 (0.15)	−0.29 (0.10)
	Progesterone	0.00 (0.12)	−0.01 (0.10)	−0.02 (0.07)	−0.16 (0.10)
	LH	0.42 (0.32)	−0.29 (0.22)	0.17 (0.33)	0.44 (0.24)
	FSH	0.01 (0.26)	0.02 (0.07)	0.27 (0.29)	0.06 (0.20)
	Testosterone	−0.00 (0.14)	0.00 (0.12)	−0.08 (0.10)	0.09 (0.14)
Blockspan backwards	Estrogen	−0.06 (0.09)	−0.02 (0.10)	−0.12 (0.12)	−0.02 (0.11)
	Progesterone	−0.01 (0.07)	−0.14 (0.10)	−0.21 (0.16)	−0.13 (0.12)
	LH	0.36 (0.50)	−0.22 (0.17)	0.52 (0.31)	0.52 (0.32)
	FSH	−0.14 (0.30)	−0.65 (0.36)	0.12 (0.19)	0.36 (0.25)
	Testosterone^a^	−0.07 (0.10)	−0.12 (0.10)	0.18 (0.09)	0.21 (0.13)
Cognitive bias	Estrogen	0.16 (0.10)	0.04 (0.07)	−0.03 (0.08)	0.05 (0.06)
	Progesterone	−0.20 (0.09)	−0.11 (0.08)	−0.08 (0.10)	0.01 (0.07)
	LH	−0.05 (0.22)	0.28 (0.16)	0.14 (0.16)	−0.04 (0.10)
	FSH	−0.21 (0.12)	0.03 (0.15)	0.00 (0.13)	−0.19 (0.08)
	Testosterone	−0.14 (0.10)	−0.06 (0.09)	−0.06 (0.13)	0.02 (0.10)
Divided attention	Estrogen	−0.12 (0.15)	0.07 (0.07)	0.01 (0.12)	0.05 (0.08)
	Progesterone	−0.11 (0.11)	−0.03 (0.11)	0.03 (0.10)	0.05 (0.08)
	LH	−0.62 (0.27)	−0.23 (0.17)	−0.30 (0.19)	0.38 (0.20)
	FSH^b^	−0.01 (0.20)	−0.12 (0.13)	0.11 (0.11)	−0.29 (0.10)^*^
	Testosterone	−0.04 (0.10)	−0.10 (0.09)	−0.19 (0.11)	0.01 (0.09)

a *Statistically significant model effect at p = 0.031*.

b *Statistically significant model effect at p = 0.010*.

* *Statistically significant parameter at Bonferroni corrected α = 0.008*.

*Statistically significant parameter estimates are highlighted in bold face*.

Changes in cognitive functioning between consecutive time-points in association with changes in estrogen, progesterone, LH, FSH, and testosterone levels are indicated in Table 4. A significant association between changes in progesterone and blockspan backwards was found from pre-ovulatory to mid-luteal phase during the first cycle (β = −0.44, *p* = 0.002). That association held when adjusted for obesity and endocrinological disorders. However, in the second cycle these parameters were unrelated (β = −0.15, *SE* = 0.15, *p* = 0.332). As for testosterone, a significant negative association with divided attention was found for intra-individual change from menstrual to pre-ovulatory (β = −0.24, *p* = 0.007) as well as from pre-ovulatory to premenstrual phase (β = −0.34, *p* = 0.007). These two associations remained unaltered when adjusted for obesity, endometriosis or PCOS, but they did not replicate using data from the second cycle (β = 0.00, *SE* = 0.17, *p* = 0.995 for menstrual to pre-ovulatory change and β = −0.20, *SE* = 0.20, *p* = 0.326 for pre-ovulatory to premenstrual change).

**Table 4:** Associations between intra-individual changes in prefrontal cognitive test scores and changes in hormone levels across the first cycle.

**Cognitive test**	**Hormones**	**Measurement**		
		**T1 thru T2**	**T2 thru T3**	**T3 thru T4**
		**β (SE)**	**β (SE)**	**β (SE)**
Blockspan forwards	Estrogen	−0.12 (0.12)	−0.18 (0.12)	−0.14 (0.15)
	Progesterone	−0.02 (0.14)	−0.10 (0.12)	0.00 (0.11)
	LH	−0.11 (0.19)	−0.10 (0.16)	0.31 (0.19)
	FSH	−0.03 (0.13)	0.10 (0.15)	0.09 (0.15)
	Testosterone	−0.17 (0.15)	−0.02 (0.15)	0.01 (0.15)
Blockspan backwards	Estrogen	−0.02 (0.10)	−0.15 (0.16)	−0.06 (0.17)
	Progesterone^a^	−0.04 (0.16)	−0.44 (0.14)^*^	0.02 (0.19)
	LH	−0.04 (0.16)	−0.01 (0.16)	0.33 (0.16)
	FSH	−0.14 (0.12)	0.14 (0.16)	0.30 (0.21)
	Testosterone	0.15 (0.14)	0.22 (0.12)	−0.04 (0.11)
Cognitive bias	Estrogen	0.14 (0.13)	0.01 (0.07)	0.02 (0.13)
	Progesterone^b^	−0.21 (0.12)	−0.31 (0.12)	−0.07 (0.14)
	LH	−0.06 (0.13)	0.00 (0.15)	0.03 (0.13)
	FSH	0.03 (0.10)	−0.03 (0.14)	−0.02 (0.13)
	Testosterone	−0.17 (0.13)	−0.01 (0.12)	−0.00 (0.08)
Divided attention	Estrogen	−0.11 (0.14)	0.06 (0.13)	0.10 (0.14)
	Progesterone^c^	−0.39 (0.15)	0.13 (0.09)	0.09 (0.13)
	LH	−0.15 (0.08)	0.16 (0.21)	0.06 (0.12)
	FSH	−0.12 (0.10)	−0.01 (0.12)	−0.04 (0.16)
	Testosterone^d^	−0.24 (0.09)^*^	−0.34 (0.12)^*^	0.01 (0.13)

a *Statistically significant model effect at p = 0.010*.

b *Statistically significant model effect at p = 0.009*.

c *Statistically significant model effect at p = 0.025*.

d *Statistically significant model effect at p = 0.001*.

* *Statistically significant parameter at Bonferroni corrected α = 0.008*.

*T1, Menstrual phase; T2, pre-ovulatory phase; T3, mid-luteal phase; T4, premenstrual phase*.

*Statistically significant parameter estimates are highlighted in bold face*.

Finally, we performed a subset analysis for healthy women only (excluding all women with obesity or endocrinological disorder). As there was no evidence for any consistent association between sex hormones and prefrontal cognitive functions across both cycles, for the sake of parsimony, here we report the results for estrogen only. In the first cycle (*n* = 46), we noted that estrogen significantly related to blockspan backward and cognitive bias (both model effects *p* < 0.001). Specifically, both blockspan backward (β = −0.59, *SE* = 13, *p* < 0.001) and cognitive bias (β = −0.50, *SE* = 10, *p* < 0.001) were negatively associated with estrogen at mid-luteal phase only. In contrast, in the second cycle (*n* = 40), estrogen did not relate to blockspan backward (model effect *p* = 0.459) and cognitive bias (model effect *p* = 0.424). With respect to intra-individual change, in the first cycle there was an association between estrogen and cognitive bias (model effect *p* = 0.004), specifically a negative association with change from mid-luteal to premenstrual phase (β = −0.42, SE = 0.13, *p* = 0.001). Again, that association did not replicate in the second cycle (model effect *p* = 0.821).

## Discussion

This multisite observational study used a stringent design including a relatively large sample size, four different measurement occasions across the cycle, statistical modeling of intra-individual change scores over time, and an attempt to replicate findings from the first cycle using data from a second cycle, to critically examine a possible association between hormone levels and prefrontal cognitive functioning, including working memory, attention and cognitive control (i.e., cognitive bias).

Most importantly, our data revealed no association between estrogen and prefrontal cognitive functions across two consecutive menstrual cycles, including between- and within-subject effects. Note that these null-associations are not a consequence of power failure, as effect sizes were consistently small (all *r* < 0.3). These results conflict with some cross-sectional findings (e.g., Hampson and Morley, 2013), but are in accord with some longitudinal observational studies in menstruating women (e.g., Mordecai et al., 2008; Griksiene and Ruksenas, 2011). Moreover, large randomized controlled trials in women making the transition to menopause (e.g., Espeland et al., 2004; Henderson et al., 2016) did not detect practically significant associations between estrogen therapy and cognitive ability in women without cognitive impairments at baseline.

We found a moderate negative association between FSH and divided attention at premenstrual phase during the first cycle, but that finding was not replicated using data from the second cycle. Moreover, this effect was not evident when specifically focusing on intra-individual change over time. The analysis of change scores is the more stringent test of a causal pathway than between-subject effects, though even this test can be confounded by random variance in hormone concentrations or systematic bias due to practice effects. More specifically, intra-individual change in progesterone levels and blockspan backwards were substantially associated from pre-ovulatory to mid-luteal phase during the first cycle, but unrelated during the second cycle. In addition, changes in testosterone were significantly related to changes in divided attention from menstrual to pre-ovulatory as well as from pre-ovulatory to mid-luteal phase. Again, these associations failed to replicate in the second cycle, which stresses how easily irreproducible false-positive findings can emerge in neuroendocrinological research (Rosmalen and Oldehinkel, 2011; Hengartner, 2017).

Another particularly striking example for inconsistent false-positive findings in small samples was provided by our subset analysis of healthy women. With respect to estrogen, we found two significant between-subejct effects during the first cycle. However, analyses using data from the second cycle did not replicate these findings. Moreower, there was evidence for one significant within-subject effect of estrogen over the course of the first cycle, but again that finding was inconsistent with data from the second cycle. Replication of anecdotal findings using data from a second cycle as implemented in the present study is therefore necessary in order to increase confidence in the reliability of neuroendocrinological findings. Another stringent approach would be to test hypotheses using randomized controlled trials. For instance, the popular “window of opportunity” hypothesis (e.g., Sherwin, 2012), which states that estrogen therapy may protect against cognitive decline only when initiated shortly after menopause, was in the meantime unequivocally refuted by a randomized, double-blind, placebo-controlled trial (Henderson et al., 2016). This trial did in fact also challange the sexual dimorphism hypothesis (e.g., Farage et al., 2008), since hormone therapy did not differentially influence verbal memory relative to placebo (Henderson et al., 2016).

Practice effects (Collie et al., 2003; Bartels et al., 2010) were observed with respect to associations between intra-individual change scores in divided attention and testosterone (see Table 4). Because divided attention improved substantially during the first half of the cycle (i.e., from menstrual to pre-ovulatory and from pre-ovulatory to mid-luteal phase) due to practice effects while testosterone remained stably low during the same time-period, our data revealed a spurious negative association between these measures of change during the first cycle. In accordance, when we tried to replicate these findings with data from the second cycle, where practicing did no longer exert an effect on divided attention (see Table 2), those artefactual associations between testosterone and divided attention disappeared. As a consequence, many published studies use counterbalanced designs to minimize confounding by practice effects. However, it is important to recognize that the specific timing of the first test application still introduces a major bias even in counterbalanced test-sequencing designs. Imagine for instance a cognitive test that is absolutely unrelated to hormone levels but whose scores are known to increases at retest due to practice effects. Now assume that half of a sample completes this cognitive test first at menstrual phase and the other three assessments consecutively at pre-ovulatory, mid-luteal and premenstrual phase, while the second group starts cognitive testing at mid-luteal phase. In the first group the practice effect runs parallel with stably low progesterone levels, while in the second group it is accompanied by a steep decrease from very high progesterone to moderately high levels during luteal phase. It comes without saying that this particular sequencing conveys a strong bias on the progesterone-cognition associations by producing a negligible positive interaction with time in the first group, but a strong negative interaction in the second. Pooled over both groups, this bias would result in a moderate negative association between cognitive ability and progesterone. Thus, obviously this effect is not counterbalanced between groups, which is why balanced sequencing of neurocognitive testing cannot eliminate practice effects.

Finally, we acknowledge the following limitations: firstly, though our sample was considerably larger than those commonly assessed in this field, a sample size of *n* > 100 would be preferable due to the substantial variance in hormone levels at given time points. Secondly, though to the best of our knowledge we were the first to use data from a second cycle as an external validation criterion, that sample consisted of a subset of women from the first cycle. Therefore, this data from the second cycle was not independent from data from the first cycle. In future research it would be worthwhile to assess an independent validation sample. Thirdly, we assessed only three cognitive functions, that is, visuospatial working memory, divided attention and cognitive bias, which are certainly not exhaustive and hence do not cover the whole range of cognitive functioning. Consideration of additional tests would be preferable with respect to coverage, but note that increasing the number of cognitive tests would also increase the probability of false-positive chance findings due to multiple testing. Fourthly, 30 women (34.1%) presented with endocrinological disorders and therefore their hormone levels may deviate from healthy controls. However, statistical control for endocrinological disorders did not alter the results and a subset analysis of healthy women did not indicate any consistent findings either. It is also worth noting that endocrinological disorders should not affect the association between hormone levels and cognitive ability, unless there would be stringent evidence that in women with endocrinological disorders hormones serve a different function than in healthy women. Finally, fifthly, we did not incorporate a counterbalanced design. Even though we believe that practice effects were ruled out in the second cycle, it could be worthwhile to combine a repeated cycle design with counterbalancing of test sequences.

## Conclusions

Our results indicate that the literature on associations between hormonal changes during the menstrual cycle and cognitive functioning is prone to inflated effect sizes and probable false-positive findings due to methodological biases and random variance. In the present study we found no consistent and meaningful associations between prefrontal cognitive functioning and fluctuations in hormone levels that replicated across two menstrual cycles, considering both inter-individual differences and intra-individual change. Regarding the inconsistencies in the published literature and the various methodological limitations in studies with positive results, we suggest that caution is warranted when conclusions are made for specific hormonal effects on cognitive functioning. The results of the present observational study based on two consecutive cycles with four assessments each and the findings of other observational studies (e.g., Mordecai et al., 2008; Griksiene and Ruksenas, 2011) as well as a recent randomized controlled trial (Henderson et al., 2016) all showed that estrogen did not relate to global cognitive functioning, including attention, cognitive control, and sexually dimorphic tasks such as visuospatial working memory or verbal fluency. To rule out confounding by practice effects and to minimize the probability of false-positive chance findings, observational studies should stringently include data from a second cycle. Moreover, more emphasis on intra-individual change in hormone levels is required, as within-subject effects have been largely neglected in this field thus far.

## Ethics statement

This study was carried out in accordance with the recommendations of Swiss Ethics with written informed consent from all subjects. All subjects gave written informed consent in accordance with the Declaration of Helsinki. The protocol was approved by the Ethics Committees of Zurich and Hannover.

## Author contributions

BL, TK, ET, TM, ME, and SR were responsible for the design and conduct of the study. KG, FI, and LS participated in data collection and data management. BL and KG participated in writing of the manuscript. MH drafted the manuscript and conducted all statistical analyses. All authors critically revised the manuscript and approved the final version.

### Conflict of interest statement

The authors declare that the research was conducted in the absence of any commercial or financial relationships that could be construed as a potential conflict of interest. The reviewer SO and handling Editor declared their shared affiliation, and the handling Editor states that the process nevertheless met the standards of a fair and objective review.
