# Supplementary material for: Lack of Associations between Female Hormone Levels and Visuospatial Working Memory, Divided Attention and Cognitive Bias across Two Consecutive Menstrual Cycles
Source: Front Behav Neurosci. 2017 Jul 4;11:120. doi: 10.3389/fnbeh.2017.00120 (PMC5495858; doi:10.3389/fnbeh.2017.00120)
Supplement: Supplementary file 1 [file Table1.DOCX]

Supplementary Table 1: Descriptive statistics of hormone assays across cycle 1 and 2.

| **Cycle 1** | **Menstrual phase** | **Pre-ovulatory phase** | **Mid-luteal phase** | **Premenstrual phase** |
| --- | --- | --- | --- | --- |
|  | N; Range, Mean (SD) | N; Range, Mean (SD) | N; Range, Mean (SD) | N; Range, Mean (SD) |
| Estrogen | 84; 57.0-425.0, 174.4 (84.2) | 82; 164.0-2322.0, 749.6 (453.2) | 80; 154.0-1227.0, 576.0 (230.5) | 72; 31.0-1227.0, 365.0 (234.8) |
| Progesterone | 84; 0.1-5.3, 1.9 (0.9) | 79; 0.7-7.6, 2.4 (1.5) | 80; 1.3-108.7, 40.0 (23.1) | 72; 0.8-69.6, 16.4 (15.8) |
| LH | 85; 2.1-16.8, 6.3 (2.7) | 69; 1.9-49.4, 13.4 (10.3) | 47; 0.8-20.6, 7.4 (5.5) | 39; 1.6-21.3, 5.3 (3.8) |
| FSH | 84; 3.7-10.4, 6.8 (1.4) | 75; 2.9-18.4, 6.8 (3.0) | 71; 1.8-10.4, 4.0 (1.7) | 45; 1.2-11.3, 4.3 (2.3) |
| Testosterone | 85; 0.0-2.6, 1.0 (0.6) | 81; 0.2-3.5, 1.3 (0.7) | 80; 0.0-2.9, 1.0 (0.6) | 73; 0.0-2.6, 1.0 (0.5) |
|  |  |  |  |  |
| **Cycle 2** | **Menstrual phase** | **Pre-ovulatory phase** | **Mid-luteal phase** | **Premenstrual phase** |
| Estrogen | 66; 69.0-463.0, 182.0 (80.3) | 67; 90.0-2223.0, 800.5 (524.7) | 67; 95.0-1168.0, 570.5 (257.2) | 52; 49.0-727.0, 313.6 (209.9) |
| Progesterone | 65; 0.5-4.1, 1.9 (0.9) | 63; 0.5-6.7, 2.4 (1.3) | 67; 1.2-99.2, 41.0 (24.2) | 51; 0.8-54.2, 11.9 (12.5) |
| LH | 67; 2.4-13.9, 6.3 (2.4) | 65; 3.8-66.4, 18.3 (15.3) | 47; 0.5-22.7, 7.0 (5.0) | 36; 0.6-15.6, 5.1 (3.1) |
| FSH | 67; 3.4-13.3, 6.8 (1.7) | 67; 3.1-14.4, 6.8 (2.6) | 58; 1.2-8.7, 4.0 (1.7) | 39; 1.5-9.9, 4.7 (2.4) |
| Testosterone | 67; 0.0-2.4, 0.9 (0.5) | 67; 0.2-2.8, 1.2 (0.6) | 67; 0.1-2.5, 0.9 (0.5) | 53; 0.1-2.7, 0.9 (0.5) |

*Note*

Unit of measure: Estrogen (pmol/l); progesterone (nmol/l); LH (lU/l); FSH (mlU/l); testosterone (nmol/l)
